# Main limitations of transpulmonary thermodilution: set targets

**DOI:** 10.1186/s13054-017-1833-8

**Published:** 2017-09-18

**Authors:** Manuel Sánchez-Sánchez, Eva Herrero, Lucia Cachafeiro, Eva Flores, Alexander Agrifoglio, Belén Civantos, Abelardo García-de-Lorenzo

**Affiliations:** 1 Hospital Universitario La Paz-Carlos III/IdiPAZ, Madrid, Spain; 2 Intensive Medicine Service, Hospital Universitario La Paz, Paseo de la Castellana 261, 28046 Madrid, Spain

## Background

We read with great interest the review by Xavier Monnet and Jean-Louis Teboul [1]. This review concluded that transpulmonary thermodilution (TPTD) provides a full haemodynamic evaluation. The authors discuss the limitations of previous studies reporting a lack of outcome improvement in patients treated using TPTD-based fluid management. They stated that the objectives of these studies were not comparable and that questionable protocols were used. Based on our previous studies of TPTD in burn patients [2], we would like to make some comments.

The TPTD technique provides parameters that must be interpreted having previous knowledge about situations which may influence results. In addition, the normal values of healthy individuals may not be appropriate comparators for critically ill patients. Therefore, the successful use of this device should be based on the achievement of appropriate objectives [3]. Extravascular lung water or the pulmonary vascular permeability index can be useful parameters for reducing the fluid load if it is elevated, but not to provide more volume if it is low because the patient may not exhibit preload responsiveness or may show only ‘temporary’ responsiveness. For instance, if there is high capillary leakage, the haemodynamic benefits obtained may be lost in a short time. Furthermore, it is erroneous to equate alteration of the pulmonary vascular permeability index with the systemic vascular permeability index [4]. In any case, this technique can help determine other parameters, such as stroke volume variation, or measure the responses of these parameters to fluid challenge.

In our study, we have found that critically ill patients with slightly low preload values (global end-diastolic volume < 600 ml/m^2^) achieve adequate cardiac index (>2.5 L/min/m^2^) and lactate values (<2 mmol/L), avoiding excessive volume contributions [2, 5].

Finally, it is important to acknowledge possible complications in certain circumstances. For example, in burn patients with severe hypovolemia we found a higher incidence of transient ischaemia in the lower limbs than the 0.4% reported in some studies.

## Conclusions

We agree with the authors that TPTD provides a full cardiovascular evaluation adequate for most critically ill patients, but we believe that in addition to improving the technique, the user requires adequate knowledge of factors that influence the measurements. We suggest that an adequate cardiac index and tissue perfusion can be achieved with below-normal levels of preload and therefore these values may be adequate in certain situations. We believe that the main limitation of this technique is the lack of accurate targets.

## Abbreviation

TPTD: transpulmonary thermodilution
